# Genetic and Epigenetic Aspects of Atopic Dermatitis

**DOI:** 10.3390/ijms21186484

**Published:** 2020-09-04

**Authors:** Bogusław Nedoszytko, Edyta Reszka, Danuta Gutowska-Owsiak, Magdalena Trzeciak, Magdalena Lange, Justyna Jarczak, Marek Niedoszytko, Ewa Jablonska, Jan Romantowski, Dominik Strapagiel, Jarosław Skokowski, Anna Siekierzycka, Roman J. Nowicki, Iwona T. Dobrucki, Anna Zaryczańska, Leszek Kalinowski

**Affiliations:** 1 Department of Dermatology, Venereology and Allergology, Medical University of Gdańsk,80-210 Gdańsk, Poland; Magdalena.trzeciak@gumed.edu.pl (M.T.); mlange@gumed.edu.pl (M.L.); rnowicki@gumed.edu.pl (R.J.N.); 2 Invicta Laboratory, Trzy Lipy 3, 80-172 Gdańsk, Poland; 3 Department of Molecular Genetics and Epigenetics, Nofer Institute of Occupational Medicine, 91-348 Łódź, Poland; edyta.reszka@imp.lodz.pl (E.R.); ewa.jablonska@imp.lodz.pl (E.J.); 4 University of Gdansk, Intercollegiate Faculty of Biotechnology of University of Gdańsk and Medical University of Gdansk, 80-307 Gdansk, Poland; danuta.gutowska-owsiak@ug.edu.pl; 5 MRC Human Immunology Unit, Weatherall Institute of Molecular Medicine, Radcliffe Department of Medicine, University of Oxford, Oxford OX3 9DS, UK; 6 Biobank Lab, Department of Molecular Biophysics, Faculty of Biology and Environmental Protection, University of Lodz, 90-231 Łódź, Poland; justyna.jarczak@biol.uni.lodz.pl (J.J.); dominik.strapagiel@biol.uni.lodz.pl (D.S.); 7 Department of Allergology, Medical University of Gdańsk, 80-210 Gdańsk, Poland; mnied@gumed.edu.pl (M.N.); jromant@gumed.edu.pl (J.R.); 8 Department of Surgical Oncology, Medical University of Gdańsk, 80-210 Gdańsk, Poland; jskokowski@gumed.edu.pl; 9 Department of Medical Laboratory Diagnostics—Biobank, Medical University of Gdańsk, 80-210 Gdańsk, Poland; asiekierzycka@gumed.edu.pl; 10 Biobanking and Biomolecular Resources Research Infrastructure Poland (BBMRI.PL), 80-210 Gdańsk, Poland; 11 Department of Bioengineering, Beckman Institute for Advanced Science and Technology, Urbana, IL 61801, USA; dobrucka@illinois.edu; 12 Department of Bioengineering, University of Illinois at Urbana-Champaign, Urbana, IL 61801, USA; 13 Dermatology Students Scientific Circle in the Department of Dermatology, Venereology and Allergology, 80-210 Gdańsk, Poland; a.zaryczanska@gumed.edu.pl; 14 Department of Mechanics of Materials and Structures, Gdańsk University of Technology, Gdańsk University of Technology, 80-233 Gdansk, Poland

**Keywords:** genetics, atopic dermatitis, skin barrier dysfunction, FLG, SPINK genes, epigenome, DNA methylation, histone modifications, micro-RNA

## Abstract

Atopic dermatitis is a heterogeneous disease, in which the pathogenesis is associated with mutations in genes encoding epidermal structural proteins, barrier enzymes, and their inhibitors; the role of genes regulating innate and adaptive immune responses and environmental factors inducing the disease is also noted. Recent studies point to the key role of epigenetic changes in the development of the disease. Epigenetic modifications are mainly mediated by DNA methylation, histone acetylation, and the action of specific non-coding RNAs. It has been documented that the profile of epigenetic changes in patients with atopic dermatitis (AD) differs from that observed in healthy people. This applies to the genes affecting the regulation of immune response and inflammatory processes, e.g., both affecting Th1 bias and promoting Th2 responses and the genes of innate immunity, as well as those encoding the structural proteins of the epidermis. Understanding of the epigenetic alterations is therefore pivotal to both create new molecular classifications of atopic dermatitis and to enable the development of personalized treatment strategies.

## 1. Introduction

Atopic dermatitis (AD) is one of the most common skin diseases affecting about 10–25% of children in industrialized countries and 7–10% of adults [1,2].

While the disease often begins early in infancy (50% within the first 6 months), the majority of cases resolve spontaneously; nevertheless, the disease may persist into adulthood and can be debilitating. Moreover, there is also a growing number of patients with late or very late AD onset. The vast majority of the patients (80%) have elevated levels of total IgE in serum and sensitization to multiple antigens, including environmental allergens and skin-colonizing microbes [3]. However, the study of published data by Flohr et al. indicates that the frequency of atopy vary from 47% to 75%. This depends on the age of diagnosis, study size studies and hospital patients examinations [4].

**Figure 1:**
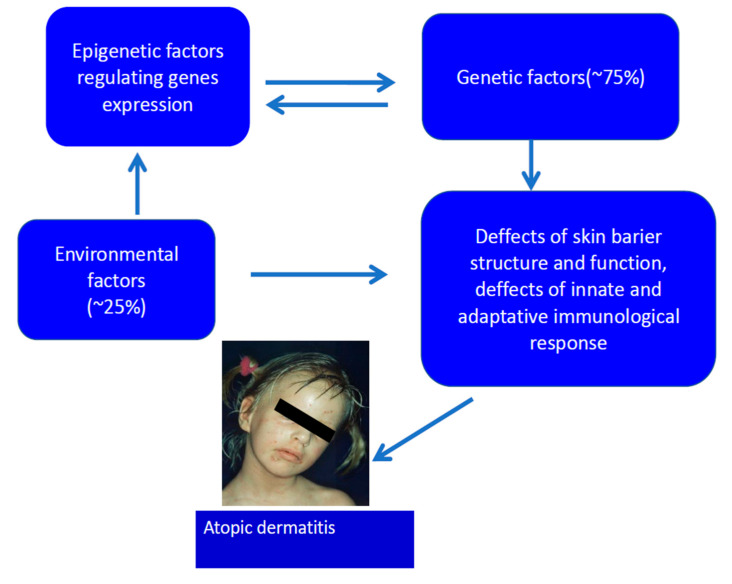
Etiology of atopic dermatitis.

Dried skin, excess water loss, and the permeable and dysfunctional epidermal barrier result as the main symptoms of patients diagnosed with AD [5,6,7,8,9,10,11,12,13,14,15,16]. The skin of the majority of AD patients is predominantly colonized by *Staphylococcus aureus*, which contributes to the pathology [17]. The etiology of AD is multifactorial and involves gene–gene and gene–environment interactions (Figure 1) [7,8,11,12,13,14,15,16]. The general view is that AD development relies on the Th2 lymphocyte-driven inflammatory responses. However, the T cell which infiltrates the skin lesions include both Th2 and Th1 cells, as well as other T cell lineages i.e., Th17 and Th22 [11,15,18,19,20]. This is in contrast to psoriasis, another persistent inflammatory skin disease, where Th1 and Th17 subsets dominate in skin [21,22].

## 2. Genetics of AD

From the genetic point of view, the disease is inherited and multifactorial; many non-allelic genes and environmental factors, which induce the symptoms, contribute to its pathogenesis [7,8,11,12,13,14,15,16,18] (Figure 1). According to recent research, over 70 genes could be associated with AD in different populations. Genes of the known contribution to AD pathogenesis can be divided into five main groups (Table 1).

The first group includes genes, mutations in which lead to the epidermal barrier dysfunction [7,8,11,12,13,16,23,24,25]. The second and third groups contain genes associated with the impairment of innate or adaptive immune response mechanisms, respectively, e.g., leading to the over-reactivity in the TLR system, the overproduction of Th2 cytokines or dysfunction in regulatory T lymphocytes; genes encoding Th1, Th17, and Th22 cytokines play essential roles in the chronic phase of the disease [1,7,8,13,14,15,26,27,28,29,30,31,32]. The fourth group includes interleukin genes produced by keratinocytes exposed to stress, e.g., UV or mechanical trauma (alarmins): IL-25, TSLP, IL-33 [7,8,13,14,17,19]. The last group contains genes implicated in the vitamin D metabolism and synthesis of its receptors [7,11,12,15]. An interesting gene is *OVOL1* (ovo like transcriptional repressor), an upstream transcription factor that regulates FLG expression. It is intriguing that *FLG*, *OVOL1* and *IL13* were the three genes most significantly associated with AD among 31 susceptible gene loci reported in a meta-analysis of genome-wide association studies [27].

However, the genetic associations described above characterize only a fraction of patients diagnosed with AD and have also been observed in healthy individuals; additionally, patients with a non-identifiable mutation also exist. This raises a question: why does the disease manifest only in some of the mutation carriers and why does it appear in patients without the mutation?

One of the main genes related to AD is the filaggrin gene (*FLG*) with null mutations observed in about half of the patients with moderate to severe AD [33,34,35,36]. Heterozygous carriers have an estimated eight-fold increased risk of the disease, while carriers of two mutated alleles are virtually always affected (with the odds ratio of > 150) [35]. The number of FLG monomer repeats in the gene sequence also influences the clinical phenotype [36] including the time of the onset and the severity of the disease. It is important to remember that *FLG* mutations as such also represent a risk factor for other atopic manifestations, e.g., asthma, suggesting that FLG deficiency may have a broader systemic significance. FLG, synthesized predominantly at the granular layer of the epidermis as a large precursor (profilaggrin; PFLG), is one of the main proteins of the *stratum corneum*. It is required for the generation of the natural moisturizing factor (NMF), which is produced upon FLG deamination and breakdown. NMF consists of amino acids (40%), pyrrolidone–carboxylic acid (12%), lactates (12%), urea (7%), Na^+^, Ca^+2^, K^+^, phosphates and chlorides (18%), glucosamine and creatinine (1,5%). NMF plays a key role in the maintenance of stratum corneum hydration and due to the mild acidity of the main compounds, also reduces its pH to about 5.5. Interestingly, besides the inherited factors, the content of FLG can be reduced by inflammatory cytokines and histamine in the atopic skin, which explains its decreased level in patients without a mutation in the *FLG* gene [6,9,10,11,12,33]. Epidermal insufficiency, resulting from one of these factors or their combination increases trans-epidermal water loss, causing the drying and cracking of the epidermis; FLG insufficiency also leads to aberrant keratinocyte differentiation, resulting in inadequate skin lipid content [37]. As a result, the insufficiency in the epidermal barrier enables the penetration of allergens and microorganisms. In addition, pH elevation results in the activation of epidermal proteases that cause uncontrolled epidermis desquamation. Proteases may also activate proinflammatory cytokines (IL-1β, IL-18, IL-33), which leads to skin inflammation [13,14,17,19]. Furthermore, several immunological mechanisms linked to filaggrin have been described, which indicate its broader immunomodulatory role [38,39,40,41].

Nevertheless, the question remains as to why some individuals, who are *FLG* gene mutation carriers or have a mutation in other AD-relevant genes, do not present symptoms of the disease [35]. To this end, data indicate that the epigenetic regulation of gene expression could be another factor in AD pathogenesis, along with the pathogenic mutations [42].

## 3. Epigenetic Regulation of Gene Expression

Epidemiological studies show that multiple environmental factors lead to the increased occurrence of AD. The most prevalent example is the lack of contact with bacterial antigens during one’s youth, referred to as “the hygiene hypothesis” [11,12,43]. What are the most likely mechanisms in which those environmental factors influence gene expression and support AD development?

We may find some answers to these questions in a new, rapidly growing field of genetics called “epigenetics”. Recently, it has been assumed that, aside from the genome, a genetic code encrypted with the DNA sequence, our cells also contain the “epigenome”, i.e., the “second code”; the superior mechanism that controls the expression of the “first code”. In the nucleus, the DNA is wrapped around nucleosomes built from alkaline proteins, histones (H1, H2A, H2B, H3, H4), which together form chromatin [7,8,13,14,41,42,43].

By the term “epigenome”, we understand chromatin modifications such as covalent modifications of histone proteins or DNA methylation and also non-coding RNA-dependent regulation. This may affect the DNA and histone proteins, and consequently, regulate the expression of genes within the genome. It should be emphasized that epigenetic changes do not modify the genetic code (i.e., DNA sequence) itself. Modifications of the chromatin structure may, however, lead to the activation or inhibition of the transcription process of certain genes, and consequently, the process of translating new mRNA into a polypeptide chain.

The completion of the human genome sequence at the end of the 20th century enabled the understanding of the encrypted code; recently, research on the human epigenome has been launched [44]. This international project aims to learn the sequences of epigenetic changes characterizing all the cells and tissues of the human organism, in both health and disease. Epigenetic changes during embryogenesis and the differentiation of cells into tissues and organs are also being explored. Those processes are mostly reversible, which gives hope that the results generated in the project will aid the development of new therapies.

A large variety of mechanisms has been identified by which epigenetic changes may regulate gene expression, including histone modification, DNA methylation, and non-coding RNA-dependent mechanisms:

a. Post-translational alterations of the histone proteins impacting chromatin architecture, affecting its density and availability for enzyme complexes (Table 1);
b. Methylation, hydroxymethylation, or the demethylation of cytosine in the regulatory gene sequences (promoter or enhancer) altering the transcription of a gene. When the DNA bases cytosine and guanine are next to each other, the silencing of particular genes may occur as a result of adding a methyl group to the DNA molecule, which results in their inactivation and prevention of transcription. On the contrary, the demethylation of the promoter triggers the transcription process and induces the expression of a particular gene (Table 1);
c. Non-coding RNA, including micro-RNAs (miRNAs), small interfering RNAs (siRNAs), long non-coding RNAs (ln-RNAs) and Pivi-interacting RNAs (piRNAs) also comprise an important signaling and regulatory tool. This affects the process of transcription, and may also alter gene expression at the post-transcriptional levels [7,11,12,16,43,44,45,46,47].

### 3.1. Post-Translational Histone Alterations

Nuclear chromatin exists in two variants; tightly packed chromatin (heterochromatin) and lightly packed chromatin (euchromatin). Because of the high compression level, heterochromatin is not accessible for enzymes participating in the transcription process; genes encoded by DNA in this state are not expressed. In contrast, in the case of euchromatin, the DNA matrix is accessible to transcriptase, and the genetic code is transcribed, so the genes located in those regions may be expressed. Chromatin compression level is influenced by post-translational modifications of histones (acetylations, phosphorylations, methylations, ubiquitinations, sumoylations). The acetylation of histones results in chromatin becoming more tightly packed which blocks transcription, while deacetylation loosens the chromatin structure, activating gene transcription in this region. Similarly, the methylation of lysine in histone H3 (Lys 9 or Lys 27) results in gene silencing, while adding a methyl group to lysine in the position 4 or 79 or to arginine in position 17 of a polypeptide chain of histone H3 leads to the activation of gene transcription (Table 2) [7,8,11,12,16].

A good example of how the regulation of gene expression at the epigenetic level works is the de-activation of the X chromosome; observed in the female cell nuclei as Barr body. The process of deactivation happens randomly and in theory, half of the cells in the female organism have a transcriptionally active X chromosome from her mother and in the other half, the one from her father. In the deactivated X chromosome, histone H4 has been shown not to be acetylated [44,45].

### 3.2. Methylation, Hydroxymethylation or Demethylation of Cytosine

The methylation of cytosine constitutes one of the most widespread epigenetic mechanisms regulating gene expression. The process is catalyzed by a specific family of enzymes i.e., DNA methyltransferases (DNA MTs). It is considered that persistently around 60–70% of cytosine bases are in the methylated state. Particular targets of the methylation process are regions of DNA where cytosine is followed by guanine, referred to as CpG sites. These sequences’ residue consist of around 70% of the proximal promoter’s regions in the human genome, constitute primary sequences and indicate the direction of transcription. The mechanism of methylation comprises a specific signaling tool that appears to restrain the addition of specific regulatory proteins to the DNA strand inhibiting the process of transcription. In contrast, cytosine demethylation catalyzed by Ten-eleven translocation (TET) enzymes triggers the promoter and stimulates the transcription of specific sequences. It has been shown that the cells of a similar function show similar gene methylation patterns [11,12,43,44,45].

**Figure 2:**
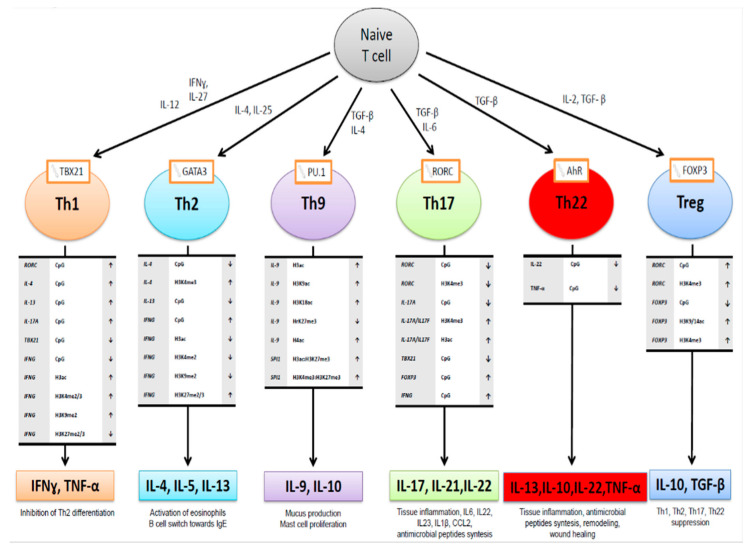
Epigenetic change in chromatin (promoter methylation, histone methylation, and acetylation) observed in the differentiation process of T helper cells subpopulations (from Potaczek et al. 2017 modified [50].

Epigenetic changes play a fundamental role in the processes of differentiation of the T lymphocytes subpopulations that are so important for the pathogenesis of AD (Figure 2). The epigenetic activation of Th2 lymphocytes producing IL-4 IL-5 and IL-13 cytokines resulting from GATA3 transcription factor activation occurs by the demethylation of *IL-13, IL-4* gene promoters and histone H3 methylation in this region as well as an increase in the methylation of *IFG* gene promoter, and decrease in H3 histone acetylation in this gene region (Figure 2). In contrast, the activation of the Th1 bias requires the methylation of the CpG islands in *IL-4, IL-13, IL17A* gene promoters, and *RORC* gene coding transcription factor, as well as demethylation of *IFG and TBX21*, and an increase in acetylation and decrease in the methylation in respective H3 histones [48,49,50]. The demethylation of the region in the *FOXP3* promoter gene, H3 residue acetylation, and methylations, accompanied by the hypermethylation of the *RORC* gene and H3 residue methylations favors the differentiation of the Th0 cells towards regulatory T (Treg) phenotype. In contrast, the methylation of the *FOXP3* promoter and the demethylation of *RORC* results in Treg deficiency, one of the features in AD pathogenesis [43,45,50,51,52].

### 3.3. Micro-RNAs

Micro-RNAs (mi-RNAs) consist of a class of small, evolutionary conserved, non-coding molecules approximately 19–25 base pair in length, single-stranded RNA, which play a vital role in regulating post-transcriptional gene expression in Eukaryotes. The fact that these RNA species remained relatively conserved evolutionary speaking indicates great biological importance. Indeed, miRNAs are involved in multiple processes such as the apoptosis, morphogenesis, proliferation, regulation of cellular metabolism, signal transduction, as well as cell differentiation [19,43,44,45,53,54].

The first product of mi-RNAs transcription is pri-mi-RNA, which is processed by the enzyme called Drosha which generates pre-Mi-RNA. mi-RNA then leaves the nucleus and is processed in the cytoplasm by the next enzyme (DICER) into a single-stranded mi-RNA. When miRNA binds to a target messenger RNA (mRNA) strand, it directly affects its stability; consequently, the complex undergoes degradation in the cytoplasm. In effect, the targeted mRNA is removed and the translation ceases which results in the inhibition of the gene function. It is considered that 1–3% of the human genome might be regulated by this epigenetic mechanism, which accounts for at least 30% of the protein-encoding genes. Interestingly, the same class of miRNA might affect the expression of several genes and vice versa, the same gene might be regulated by different miRNA species [19].

Recently, there has been growing interest in the miRNA-based diagnostics from body fluids. These small molecules can be detected in plasma, tissue fluid, milk, and urine, and may be contained within exosomes, small transport liposomes which could affect other cells. miRNA seems to be highly resistant to environmental factors. Interestingly, miRNA content in the fluids may carry a function; e.g., studies demonstrated that the presence of miRNA in exosomes of human breast milk may affect the immune system of a newborn child [19,53,54,55,56,57,58,59,60].

### 3.4. Non-Mendelian, “Heretic” Transgeneration Inheritance of Epigenetic Changes

Interestingly, some studies suggest that epigenetic changes could be involved in transgenerational inheritance; i.e., epigenetic patterns may be inherited by the progeny. For example, chemically modified DNA or the chromatin of reproductive cells contribute to the expression of a single allele in the progeny, which is called “the phenomenon of genome staining” (or “genomic imprinting”) [61,62,63,64,65]. Genomic imprinting represents yet another example of non-Mendelian inheritance, which could be called “heretic inheritance” [66]. As in conventional inheritance, genes for a given trait are passed down to the progeny from both parents. However, these genes are epigenetically marked before transmission, altering their levels of expression. These imprints are created before gamete formation and are erased during the creation of germ line cells. Therefore, a new pattern of imprinting can be made with each generation. We can call this transmission “heretic” as other non-mendelian traits (extranuclear, mitochondrial heredity, infections heredity, post zygotic recombination). Certainly, epigenetic modifications of germline cells can be carried into cellular clones during embryogenesis and organogenesis, as well as furring the differentiation of functional subsets of immune cells (Th1, Th2, Th17, Th22, and Treg lymphocytes); hence, the cells of a given tissue may inherit a specific pattern of a chemical modification of DNA and chromatin. This epigenetic modification pattern undergoes adjustments in the process of maturation and differentiation of the cells of the immune system, during prenatal development and ontogenesis under the influence of environmental factors [7,8,13,14,41,42,43,60].

## 4. Epigenetic Changes in Atopic Dermatitis

Recent data indicate that the profile of epigenetic changes in patients suffering from AD differs from that seen in healthy individuals. This applies to the genes affecting the regulation of immune response and inflammatory processes, e.g., the inhibition of responses dependent on Th1 lymphocytes, and the promotion of the Th2 bias, as well as the genes involved in innate immunity and those encoding the structural proteins of the epidermis [7,8,13,14,40,61,62,63,64,65,66]. In the skin, it has been shown that DNA methylation profiles also differ in the epidermis of AD patients in comparison to those in healthy people; specifically, this involves genes of known structural and antimicrobial function, i.e., *S100* or keratin genes (Table 3). In addition, the OAS cluster, encoding enzymes required for the synthesis of adenosine receptors involved in the activation of specific antiviral RNAses (oligoadenylate synthase proteins) are also affected. These genes are regulated by interferon and participate in the innate immune defense [67]. Furthermore, the demethylation of the *TSLP* promoter was observed in keratinocytes isolated from AD patients resulting in the overexpression of this Th2-biasing alarmin in their epidermis [68]. Similarly, the demethylation of the promoter of *FCER1G* (encoding high-affinity IgE receptor, FcεRI) was observed in monocytes of the AD patients resulting in its overexpression on the surface of these cells [69]. In addition, the *KIF3A* gene has recently been shown to be involved in skin barrier function. Disease-associated KIF3A variants alter gene methylation and expression impacting skin barrier and atopic dermatitis risk [70,71,72,73,74].

Atopy was found to have evidence of imprinting on chromosomes 3, 6, 11, 14, and 13. The β subunit of the IgE receptor on chromosome 11q12-13 may be maternally imprinted [63,64], and the locus of AD from 3q21 is paternally imprinted [65].

As highlighted above, mutations leading to reduced filaggrin expression are the major factor contributing to AD pathogenesis. Studies by Ziyab et al. have shown that the excessive methylation of this gene may occur in heterozygotic carriers of null mutations (R501X, 2282del4, and S3247X); the interplay between the sequence and epigenetic regulation results in the increased risk of the disease in the carriers. These studies indicate that the epigenetic mechanisms regulating the expression of the *FLG* gene in the pathogenesis of the disease should not be overlooked [42].

Alterations in the expression of several specific miRNAs are also observed in the skin and /or the serum of AD patients are presented in Table 3. These are involved in the regulation of expression of genes which determine Th2 polarization, the function of regulatory T lymphocytes, inflammatory processes, tight junction formation, epidermal keratinocytes proliferation and apoptosis, synthesis of cytokines and chemokines [55,56,70,71,72,75]. A study by Sonkoly et al. [70] showed that the expression of 10 miRNA species (e.g., miR-21, miR-146a, miR-155, miR-223) is increased within AD skin lesions; at the same time, the expression of 34 miRNAs (including Let a-d, miR-365, miR-375, miR-375, and miR-193c) was decreased.

Similarly, miR-146a inhibits the IFNγ-inducing events, such as the activation of TLR receptors by bacterial lipopolysaccharides as well as NFκB associated with the activation of the respective intracellular signaling. Moreover, miR-146 directly blocks the expression of the *TRAF6, IRAK1, IRAK2,* and *RELB* genes regulating the activation of this pathway. The transcription factor NFκB controls the expression of many genes required for the proinflammatory cytokine footprint, i.e., *IL1B, TNFA, CARD10, IRAK1*, chemokine genes: *CCL5* and *CXCL8* (encoding IL-8), and the miR-146a itself. Inhibiting the expression of those genes decreases the effectiveness of the innate antimicrobial response, which is a feature of AD. MiR-146a also blocks the expression of the *STAT1* gene in the regulatory T cell subset, which promotes Th1 by the suppression of Treg [19,56,72].

Sonkoly et al. demonstrated that miR-155 was one of the most up-regulated miRNAs in AD patients. In particular, miR-155 is necessary for the differentiation of T helper type 17 (Th17) and Treg cells [55,78]. The miR-155 molecule inhibits the expression of CTLA-4 (cytotoxic T-lymphocyte-associated protein 4) molecule, an immune checkpoint receptor that has an inhibitory effect on T cell responses. Hence, the increase in the miR-155 expression in the AD skin results in a loss of the control mechanism dependent on Tregs, which leads to an increase in effector T cell proliferation and results in sustained inflammation [11,12,19,43,55,56,57,58,70]. Ma et al. demonstrated that miR-155 was overexpressed in AD patients and positively correlated AD severity. In addition, the percentage of Th17 cells was increased in AD patients and there was a positive correlation between miR-155 expression and Th17 cell percentage [79].

The miRNA molecules from the Let-7 a-d family were the inhibitors of IL-13 and CCR7 synthesis. Hence, the decrease in Let-7 a-d expression observed in the atopic skin promotes the overproduction of this cytokine and contributes to the Th2 bias [56]. Similarly, miR-375 molecule, which induces the synthesis of TSLP (by blocking the expression of a transcription factor KLF5) and enhances Th2 responses in the skin [19].

Loss of hsa-miR-26a-5a is associated with the increased expression on AD skin hyaluronian 3 synthase (HAS3) involved in synthesis hyaluronian acid, a major component of extracellular matrix [76].

MiR-151a level was significantly higher in the plasma of AD patients as compared with the healthy individuals. Chen et al. showed that miR-151a was involved in the pathogenesis of AD by regulating IL-12 receptor β2 (IL12RB2), a subunit of IL-12 receptor. Overexpressing miR-151a in human T helper cells significantly downregulated IL12RB2 expression [79].

Studies in mice indicate that miRNA therapy could become one of the therapeutic strategies for AD. For example, Yoon et al. [80] targeted CCL22, a chemokine influencing the recruitment of Th2 lymphocytes expressing cutaneous lymphocytes antigen (CLA) into the skin. The study showed that the dietary administration of *Salmonella typhimurium* expressing CCL22-targeting miRNA blocked the expression of CCL22 in vivo, which led to differences in the severity of AD lesions, pruritus and IL-4, and CCL22 levels.

miR-151a and hsa-mir-144-3p have been proposed as potential biomarkers of AD [81,82]. The increased expression of Hsa-mir-144-3p has been observed in umbilical cord serum, and a high level of MiR-151a were observed in the serum of AD patients [79,80]. miR-151a would reduce IL12RB2 levels in T-cells, favoring the increase in Th2 cells.

## 5. Epigenetic Regulation of Gene Expression in Pregnancy and Early Childhood

**Figure 3:**
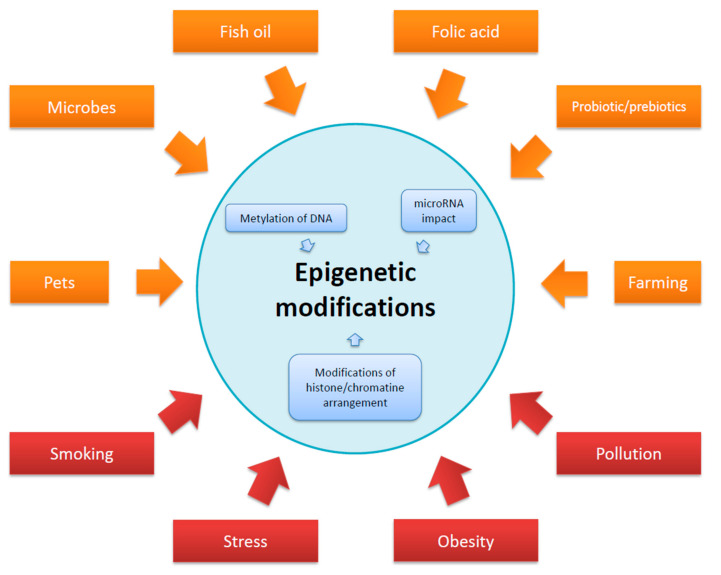
Factors acting on the body of a pregnant woman and affecting the epigenome of a child. All factors could have a modifying effect.

It is regarded that the epigenetic regulation in pregnancy could affect the well-being of a newborn, influencing the epigenome of the offspring. A number of different factors acting on the pregnant mother and in early childhood that alter the epigenome are presented in Figure 3.

Fetal CD4+ lymphocytes vary from those the adults in the hypermethylation status of the *IFNG* gene promoter; this leads to the inhibition of Th1 differentiation [83]. Changes in chromatin conformation (heterochromatinization) were also observed. As such they reduce the availability of the transcription enzymes to the *TBX21* locus. The transcription factor TBX21 plays a major role in the differentiation of the Th1 lymphocytes [84]. Interestingly, a significantly lower number of Treg lymphocytes is seen in the fetal blood than in the adult blood; this may promote increased Th2 cell responsiveness [85,86].

Epidemiological studies indicate a relationship between the disturbances in commensal bacterial composition and increased allergy risk; it also correlates with an early onset of the symptoms. In agreement with this, children who are treated with antibiotics in early childhood have a higher risk of allergies [87,88]. There is a lack of direct data on the impact of the microorganisms on the epigenetic processes in allergy, however, some data indicate that the alterations in function and composition of the gut microbiota are implicated in the pathogenesis of metabolic diseases, e.g., obesity and diabetes via the induction of epigenetic changes, such as DNA methylation, histone modifications and regulation by non-coding RNAs [89].

For atopic dermatitis, it has been indicated that higher frequencies of Treg lymphocytes were observed in the umbilical blood of children born from mothers in rural areas, and their functions were more pronounced [90,91] than of those from urban areas. This may also be compared with the observed demethylation of the FOXP3 gene promoter in the children of women drinking raw cow’s milk during their pregnancy [90]. Similarly, feeding children with raw cow’s milk seems to increase the demethylation of the FOXP3 gene in the peripheral blood cells and the Treg numbers [90,91]. Comparisons of the neonatal methylation profiles of cord blood cells between the individuals living in the rural vs. urban areas also showed the hypomethylation of *ORMDL1* (ORM1-like protein 1, endoplasmic proteins associated with sphingolipid metabolism) and STAT6. In addition, the hypermethylation of the *RAD50* (encoding DNA repair protein RAD50, associated with the replication and DNA repair) and IL13, was also observed in the former. This favors a reduction in the IL-13 synthesis and Th2 lymphocyte activity [92,93], beneficial in terms of allergy prevention. Studies in mice have demonstrated that the exposure to microorganisms may influence gene expression regulation and promote the Th1 response. For example, the prenatal administration of Gram-negative bacteria, *Acinobacterium lwoffi F78* results in the acetylation of histone H4 in the IFNG locus in mice and as a consequence, an increase in the IFNγ synthesis [94].

Epigenetic changes in the genome may be also linked to the exposure to polycyclic aromatic hydrocarbons, such as benzo(a)pyrene in tobacco smoke and polluted air [95,96,97]. Cigarette smoking by pregnant women results in the epigenetic changes that promote early onset of atopy in the child [83,84,85,86]. Specifically, the decreased intensity of the methylation process of the TSDR regulatory region in the *FOXP3* gene was observed in the fetal cells; this was associated with a decrease in the number of Tregs in the umbilical cord blood and significantly increased the risk of developing AD and food allergy before the age of 3 [95,96,98]. Cigarette smoking during pregnancy also resulted in an increase in expression of miR-223 in the umbilical blood, correlating with a decrease in the Treg number and increased AD risk before the age of 3. Moreover, miR-223 inhibits *IGF1R* (insulin-like growth factor 1 receptor) affecting the expression of the IGF receptor, which plays a role in the regulation of cellular metabolism, cell proliferation, and apoptosis [99]. The exposure of a pregnant woman to polycyclic aromatic hydrocarbons contained in the polluted air seems to be also associated with an increase in the methylation of the *IFNG* promoter in the umbilical cord blood cells and a decrease in its expression, which, again, promotes the development of Th2-dependent allergic reactions as shown above [97].

## 6. Therapies of AD

AD is regarded as a chronic, persistent disease; therefore, the treatment strategy of AD should be focused on symptomatic therapy, inflammation control and maintaining the remission period. The therapy should however be individualized regarding patients’ characteristics and course of the disease.

The local treatment is considered the mainstay of AD management. First step is education, the avoidance of clinically relevant allergens and emollient therapy [100,101].

The emollients may be the only one strategy or added to topical anti-inflammatory drugs or systemic treatment. It is worth emphasizing that adequate moisturizing reduces the risk of AD exacerbations as well as the necessity of the implementation of steroid treatment [100,101]. Emollients are recommended twice daily, ca 250 g/weekly for adults. Bathing or showering itself should include using lukewarm water as well as nonirritating, pH-balanced skin care products [100,101].

Topical anti-inflammatory treatment based on topical corticosteroid (TCS) and calcineurin inhibitors (TCI). TCS, in most cases of class II and III, according to Niedner, are used in the acute stage for a short period of time, e.g., 3–5 days and followed by TCI [100,101]. The latest are especially useful in sensitive skin areas and occlusive areas. Proactive treatment with tacrolimus helps to control the disease [102]. Wet wrap therapy with properly diluted TCS is relatively safe and a sufficient therapeutic strategy in severe, recalcitrant AD in children [103].

Patients with AD are more vulnerable to bacterial skin infections, especially with staphylococcal bacteria. However, antibiotics should be limited to only the exacerbations with clinical signs of bacterial infections to avoid the risk of sensitization or bacterial resistance [100,101].

In the case of moderate to severe cases of AD, topical therapy should be usually accomplished by phototherapy or systemic immuno-supressive/immunomodulatory treatment. Cyclosporin A is recommended as first lime treatment in such cases. Oral corticosteroids should be limited to short treatment periods [101,102]. As an alternative, methotrexate, azathioprine and mycophenolate mofetil may be also considered [101,102]. Currently, new targeted treatment strategies in managing AD are being developed. The first biologic agent ant- IL-4 receptor monoclonal antibody was registered for AD treatment [102,104]. The trend for personalized medicine appears viable.

## 7. Therapies of AD with Drugs Interfering Epigenetic Processes

To date, there are no data on therapies with drugs interfering with the epigenetic processes in atopic dermatitis. In contrast, in leukemia treatment, specific compounds that inhibit the activity of methyltransferases among others have already been introduced to clinics [105,106,107]. In line with that, Steelant et al. suggest that the introduction of therapies that deliberately interfere with epigenetic status in allergic rhinitis should be concurrent to the scientific advancement [108]. What is worth emphasizing, glucocorticosteroids which are in extensive clinical use have properties reducing the activity of histone deacetylase (HDAC), one of the key enzymes involved in the epigenetic processes [109]. Moreover, in an animal model of this disease, trichostatin A histone deacetylase inhibitor can selectively diminish the expression of T2-type cytokines by reducing, thereby worsening, the symptoms of atopic dermatitis (AD) [110]. The authors also noticed the positive effects of the use of a demethylase inhibitor H3K27me3 for asthma prevention in an animal model [111]. The loss of miR-335 is observed in AD skin. Recently, Liew et al. demonstrated that Belinostat, the inhibitor of histone deacetylase, restores epidermal miR-335 expression and rescues the defective skin barrier in AD by targeting the dysregulated miR-335/SOX6 axis [111].

However, to develop an effective atopic dermatitis and allergy-directed therapy acting through the deliberate modification of genes responsible for type Th2 bias at the epigenetic level requires a much better understanding of the complex mechanisms and dependencies; this will hopefully be seen in the coming years [107,108,109,110].

## 8. Summary

In summary, AD is a heterogeneous disease in which the interplay between genomic changes associated with mutations in the key barrier and immune genes and a spectrum of environmental factors play a fundamental role in the pathogenesis. To make it more complex, recent studies indicate the importance of epigenetic alterations in the development of the disease. Epigenetic modifications are mainly mediated by DNA methylation, histone acetylation, and the action of specific micro-RNAs. It has been determined that the epigenome in AD patients differs from the one observed in healthy individuals. This applies especially to the genes regulating immune responses and inflammatory processes, i.e., those involved in the Th1 inhibition and Th2 bias, genes of the innate immunity as well as those encoding the structural proteins of the epidermis. A thorough understanding of the epigenetic modifications may provide a basis for new molecular classifications of the disease and the development of personalized therapies.

## Figures and Tables

**Table 1:** Main groups of genes associated with atopic dermatitis (AD) pathogenesis [5,6,7,9,10,12,23,24,25,26,27,28,29,30,31,32].

Pathological Process in AD	Example of Genes Involved
Epidermal barrier genes	*Filaggrin, filaggrin 2, hornerin* Corneodesmosomal genes (*desmoglein, desmocollin*) and tight junction genes (*claudins, ocludins*) Epidermal protease genes (*kallikreins, cathepsins, caspase 14*), and their inhibitors (*SPINK5, Cystatin A)* *OVOL1* (ovo like transcriptional repressor)—transcription factor that regulates FLG expression
Genes of the innate immune mechanisms	*TLR1, TLR2, TLR4, TLR6, TLR9, TLR10, CD14, NOD1* and defensins (*DEFB1*)
Genes of the adaptive immune mechanism	Genes of receptor subunits for IgE (*FcεRI α i FCεRI-¥)* Genes of Th2 response: *IL-4, IL-5, IL-13, IL2RA, IL-13RA IL-5RA*, *TSLPR*, *IL-4R*, *IL-18*, *IL-31* Other genes of Th bias *IL17A, TNFα, IL-22* Treg genes: *STAT-6, FOXP3, LRRC32*
Genes encoding alarmins produced by keratinocytes	*IL-25, TSLP, IL-33*
Genes regulating DNA methylation	*KIF3A*
Genes regulating vitamin D pathways	*CYP27A1, CYP2R1, VDR*

**Table 2:** Examples of the epigenetic changes of chromatin and their influence on gene activity (from [11], modified).

Chromatin Modification			Activation of Gene Transcription	Deactivation of Gene Transcription
Promoter methylation				
Methylation of cytosine in gene promoter			no	yes
Demethylation of cytosine in the promoter			yes	no
Histone modification				
Acetyla-tion of histone amino acids residues	H2A	K5*	yes	no
	H2B	K5,K12,K15,K20	yes	no
	H3	K4,K14,K18,K23,K27	yes	no
	H4	K8, K16	yes	no
Methy-lation of histone amino acid residues	H3	K4, K79, R17	yes	no
	H3	K9, K27	no	yes
	H4	R3	yes	no
	H4	K20	no	yes
Ubiquiti-nation of histones	H2A	K119	no	yes
	H2B	K120	yes	no

* K—lysine residue, R—arginine residue.

**Table 3:** miRNA change in atopic dermatitis (data from [7,11,53,75,76,77,78]).

miRNA Expression Change in AD Lesions or Serum	Associated Effect
↓Let-7 a-d	↑IL-13↑CCR7
↓miR-375	↑TSLP (Thymic stromal lymphopoietin)
↓hsa-miR-26a-5a	↑HAS3 (hyaluronian 3 synthase)
↑miR-21	↓IL12
↑miR-29b	Promotion of INF-γ-induced keratinocyte apoptosis
↑miR-146a	↓STAT1 and decrease in Treg activation ↓NFκB–pro-inflammatory transcription factor
↑miR-155	↓CTLA-4 and decrease in Treg proliferation ↑Promotion of Th17 differentiation ↓Inhibition of tight junction formation
↑miR-223 in umbilical cord blood	Decrease in Treg activation
↑MiR-151a in serum	Inhibition of IL-12 signaling
Other miRNA expression changes in atopic skin: ↑ miR-17-5p, ↑ miR142-3p/5p, ↓ miR-122a, ↓miR-326, ↓miR-133b, ↓miR-125b, ↓miR375, ↓ miR193c, ↓miR365	
